# Tackling the Electro-Topography of the Selves Through the Sphere Model of Consciousness

**DOI:** 10.3389/fpsyg.2022.836290

**Published:** 2022-05-19

**Authors:** Patrizio Paoletti, Rotem Leshem, Michele Pellegrino, Tal Dotan Ben-Soussan

**Affiliations:** ^1^Research Institute for Neuroscience, Education and Didactics, Patrizio Paoletti Foundation, Assisi, Italy; ^2^Department of Criminology, Bar-Ilan University, Ramat Gan, Israel

**Keywords:** consciousness, self, electrophysiology, meditation, executive functions, EEG, frequency

## Abstract

In the current hypothesis paper, we propose a novel examination of consciousness and self-awareness through the neuro-phenomenological theoretical model known as the Sphere Model of Consciousness (SMC). Our aim is to create a practical instrument to address several methodological issues in consciousness research. We present a preliminary attempt to validate the SMC *via* a simplified electrophysiological topographic map of the Self. This map depicts the gradual shift from faster to slower frequency bands that appears to mirror the dynamic between the various SMC states of Self. In order to explore our hypothesis that the SMC’s different states of Self correspond to specific frequency bands, we present a mini-review of studies examining the electrophysiological activity that occurs within the different states of Self and in the context of specific meditation types. The theoretical argument presented here is that the SMC’s hierarchical organization of three states of the Self mirrors the hierarchical organization of Focused Attention, Open Monitoring, and Non-Dual meditation types. This is followed by testable predictions and potential applications of the SMC and the hypotheses derived from it. To our knowledge, this is the first integrated electrophysiological account that combines types of Self and meditation practices. We suggest this electro-topographic framework of the Selves enables easier, clearer conceptualization of the connections between meditation types as well as increased understanding of wakefulness states and altered states of consciousness.

## Introduction

For centuries, scientists and philosophers have been tackling the question of what consciousness is, in order to understand what underlies human behavior, feeling, and action (Barrett and Satpute, 2013). Consciousness is a volatile phenomenon that is not easily defined, yet it determines our perception of what we consider as objective reality. While most operational definitions offer some attempt to characterize its various aspects, such definitions do not give a completely satisfying account of the way we know we are aware of our self and our existence (for a detailed argument, see Ardila, 2016). Consciousness is often regarded as consisting of two main components: awareness (the “content” of consciousness), and wakefulness or arousal (the “level” of consciousness). In addition to perception of the events of the “outside world,” awareness also refers to the subjective experience of internal phenomena: our perception of inner “events,” whether or not related to external ones, that comprises our subjective reality (Vithoulkas and Muresanu, 2014). This may also include self-awareness, i.e., awareness of one’s internal world of thoughts, reflections, imagination, and emotions as an expression of the Self. There is also a more elaborate level of self-awareness involving higher-order cognitive mechanisms such as attentional focus on one’s own mental states that is considered a distinct form of reflexive awareness (Peters, 2009), or “self as context” (Grieger, 1985).

Neuroscientific research has adopted different notions of the Self, the most frequently used of which is the binary distinction between the *Narrative Self* (NS) and the *Minimal Self* (MS) (James, 1890/1950; Gallagher, 2000; Legrand, 2006, 2007; Zahavi and Gallagher, 2008; Paoletti and Ben-Soussan, 2019, 2020; Gallagher and Zahavi, 2020; Paoletti et al., 2020). In the SMC, a third state is added, called *Overcoming of the Self*, indicating the complete absence of any sense of self (Paoletti, 2002a,b; Paoletti and Ben-Soussan, 2019, 2020; Paoletti et al., 2020). In the last decade, each of these three selves has been regarded in contemplative neuroscience as a particular configuration of self-awareness and mental contents (Gallagher, 2000; Jerath et al., 2015; Sugimura et al., 2021). There have also been attempts to link self-states to higher-order cognitive functions (i.e., executive functions), although the existing research is still scant (Ardila, 2016; Wade et al., 2018). Nonetheless, there is some evidence suggesting that the Self can be examined as a multidimensional construct denoted by: (1) First-person subjective reporting–such as the self-awareness experience; (2) Behavioral characteristics expressed through executive functions; and (3) Third-person objective measurements, such as electrophysiology (Fingelkurts et al., 2020). Accordingly, several theoretical models of selves have been proposed based on first-person phenomenology (James, 1890/1950; Gallagher, 2000; Legrand, 2006, 2007; Zahavi and Gallagher, 2008; Paoletti and Ben-Soussan, 2019, 2020; Fingelkurts et al., 2020; Gallagher and Zahavi, 2020; Paoletti et al., 2020); however, the challenge of empirically examining and validating them remains.

In this theoretical paper, we propose a novel examination of consciousness and self-awareness through the lens of the neuro-phenomenological theoretical model known as the Sphere Model of Consciousness (SMC; Paoletti, 2002a,b; Paoletti and Ben-Soussan, 2019, 2020; Paoletti et al., 2020). Although there are methodological challenges involved in examining and validating this model, we suggest ways to overcome these challenges through an “electro-topography of the Selves” that depicts cognitive and electrophysiological correlates of the different states of the Self, induced by different meditative practices. Of course, the subjective phenomenology of first-person experience remains an indispensable part of any consciousness research (Varela, 1996; Depraz et al., 2000). Thus, our aim is to create a practical instrument bridging first person reports and electrophysiology, in order to resolve some of the methodological challenges of consciousness research, as outlined below.

**FIGURE 1:**
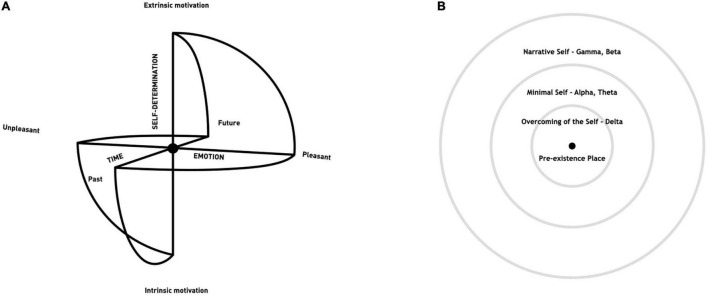
**(A)** The Sphere Model of Consciousness. **(B)** Different Selves in the SMC and their main suggested electrophysiological correlate. The Narrative Self is related to Gamma and Beta, the Minimal Self is related to a shift toward Alpha and Theta (Carhart-Harris et al., 2014), and Overcoming the Self is mostly related to Delta activity (Dor-Ziderman et al., 2013; Paoletti et al., 2020). Adapted from Paoletti and Ben-Soussan (2019, 2020).

First, we will show how each state of the Self connects with a particular type of self-awareness and first-person phenomenology of experience, and then link these to executive functions. Second, by isolating some of the specific electrophysiological and neuronal correlates of each Self and meditative practice, we will suggest that hierarchies of meditation and the Selves (Laukkonen and Slagter, 2021) share a similar electro-topography. As the hierarchical electrophysiological correlates of Selves have yet to be conceptualized within one unified model, we will detail the electrophysiology of the Selves, followed by the electrophysiology of meditation and each specific Self, all within the framework of the Sphere Model of Consciousness (SMC) (Figure 1A; Paoletti, 2002a,b; Paoletti and Ben-Soussan, 2019, 2020; Paoletti et al., 2020).

## The Sphere Model of Consciousness, Hierarchy of Selves, and Executive Functions

The three Selves conceptualized in the SMC are: the Narrative Self, the Minimal Self, and the Overcoming of the Self (SMC; Paoletti, 2002a,b; Paoletti and Ben-Soussan, 2019, 2020; Paoletti et al., 2020; see Figure 1A). They are represented as concentric circles around a center (Figure 1B), with the Narrative Self (NS) on the periphery, the Minimal Self (MS) in the middle, and Overcoming of the Self (OCS) in the center.

Each Self is characterized by a particular state of self-awareness and first-person phenomenology that are expressed through features driven by functionality and cognition. In turn, the specific features of each state of the Self facilitate regulatory processes supported by executive functions (EFs) (Barkley, 2001; Nigg, 2017), as we will see shortly. The Narrative Self is considered as a self-image built through autobiographical memories and projections into the future; it involves awareness of personal identity and its continuity through time, as well as conceptual contents (Oatley, 2007). The Minimal Self emerges from the awareness of a situated living body as a sensorimotor unit that enables selfhood in the physical world in the “here and now” (James, 1890/1950; Gallagher, 2000), has a short temporal extension, and is endowed with a sense of action, property, and first person non-conceptual content. In our previous papers about the SMC, we specified the addition of a third state, called Overcoming of the Self (see Figure 1B), in which all sense of self disappears yet subjective experiences are still able to be experienced and eventually reported (Paoletti, 2002a,b; Paoletti and Ben-Soussan, 2019, 2020; Paoletti et al., 2020).

As we have said, each of the above-mentioned Selves is linked to core self-awareness features driven by functionality and cognition, and they facilitate regulatory processes supported by executive functions (EFs) (Barkley, 2001; Nigg, 2017). EFs are a set of higher-order cognitive functions often linked to the prefrontal cortex of the brain, consisting of three key components–working memory, inhibition, and cognitive flexibility–from which more complex and higher-order EFs are built (e.g., reasoning and planning) (Miyake et al., 2000; Diamond, 2013). EFs are essential for controlling unregulated behaviors, whether external (e.g., physical actions), as well as internal (e.g., meditative practices) (van Gaal et al., 2009; Hofmann et al., 2012). Furthermore, effective self-regulation processes require self-awareness of one’s actions, thoughts, and feelings as well as the ability to make desired changes when needed (Heatherton, 2011). The multidimensional nature of EFs creates complexity regarding how they are conceptualized theoretically (Anderson, 2002; Welsh et al., 2006). This complexity is evident in the examination of latent components of EFs (e.g., shifting, inhibition) in relation to other multidimensional constructs, such as Self and awareness. The convergent and discriminant validity of these constructs is not clear, and the nature of their differences remains to be determined.

The different Selves–Narrative Self, Minimal Self, and Overcoming of the Self–may involve different usages of EFs. As we have seen above, the Narrative Self, depicted toward the periphery of the SMC sphere, is a more or less coherent self (or self-image) that is constituted of a past and a future in the various narratives that we and others tell about ourselves. It relies on declarative and episodic memories of the past or projection into the future (Vago and David, 2012) and, for an evaluative and personal perspective to be constituted and maintained over time, executive and volitional processes are necessary (Bortolan, 2020). Indeed, narrative abilities required to establish the Narrative Self are associated with core components of EFs and the higher-order cognitive functions that emerge from them, such as working-memory, inhibition, and set-shifting (Friend and Bates, 2014). However, once the Narrative Self is constructed and established, one can act more automatically with little forethought based on accumulated and internalized learning and experience. Thus, in the Narrative Self, a person can operate with relatively low self-awareness.

In contrast, the Minimal Self, depicted between the periphery and the center of the SMC sphere, is usually connected to conscious experience and a specific kind of self-awareness in which experiences are assimilated immediately as one’s own experiences without need for any inferential processes (Lane, 2020). This form of self-experience in the Minimal Self is connected with pre-reflective self-consciousness (Bortolan, 2020). As Gallagher and Zahavi (2020) explain, on the one hand, self-experience is “non-observational” because it does not depend on any kind of introspective attitude taken by the subject toward the experience itself. On the other, it is “non-objectifying” because through it the self is not treated as an object, but rather as the subject of a conscious state. It can be claimed that in the Minimal Self state, a person deliberately reduces the use of executive processes and at the same time increases the level of bodily self-awareness.

Finally, as the level of consciousness of the Self approaches the center of the SMC sphere, toward the Overcoming of the Self, the necessity of EFs may decrease considerably, or even cease completely.^1^ It is also entirely possible that the use of EFs in different Selves may not only vary in how *much* they are involved but also in the *way* they are involved. For example, a recent review (Gallant, 2016) highlighted a specific, instead of general, improvement in inhibition following mindfulness meditation, but showed inconsistent benefits in memory and cognitive flexibility, the other two main components of EFs (Miyake et al., 2000).

## Toward an Electro-Topography of the Selves

In the last few years, there have been several attempts to link different features of self-awareness (e.g., identity, witnessing) with their electrophysiological correlates (Raffone et al., 2019; Fingelkurts et al., 2020; Sugimura et al., 2021), indicating a noticeable interest of neuroscientific research in finding a method to correlate first-person phenomenology and objective measures. One such attempt at a three-dimensional construct model for complex experiential selfhood was proposed, focusing on cortical alpha activity (Fingelkurts et al., 2020). It suggests that increased frontal alpha connectivity during the experience of a “*non-symbolic, non-linguistic sense of self-presenting being*,” such as a sharper, more vivid experience of being a witnessing agent for oneself, possibly reflects being more conscious and able to access the external world, as well as the first-person perspective (Fingelkurts et al., 2020). Furthermore, temporal dynamics reflected in the brain’s electrophysiological activity have been linked by Sugimura et al. (2021) to another core feature of self-awareness, namely, the sense of consistency of self. In turn, the subjective sense of an integrated and coherent Self across time, conceptualized as identity (Erikson, 1968), has been correlated to better cognitive performance, as well as to enhanced moral judgment and creative problem solving (Dietrich and Kanso, 2010; Travis et al., 2011; Charles et al., 2014).

Models of the self, such as those proposed by Fingelkurts et al. (2020) and Sugimura et al. (2021) focus mostly on alpha cortical sources. Yet, contemplative studies, which serve as excellent means of examining the states of Self, have also emphasized the importance of additional bands as well (Travis et al., 2010; Glicksohn and Berkovich-Ohana, 2011, 2012; Kerr et al., 2011; Muthukumaraswamy et al., 2013; Flor-Henry et al., 2017; Wahbeh et al., 2018; Glicksohn and Ben-Soussan, 2020). Taking this into consideration, we suggest the inclusion of additional frequency bands in order to find more comprehensive electrophysiological marker evidence differentiating the different Selves.

**FIGURE 2:**
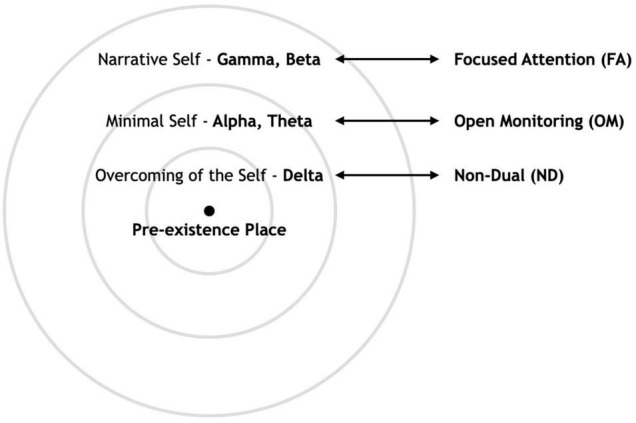
The hierarchy of Selves in the Sphere Model of Consciousness corresponding to the hierarchy of meditation and their primary electrophysiological correlates.

Toward this aim, we present a preliminary attempt to integrate into the SMC a simplified topographic map of the Selves by band, in which the NS is related to higher frequency bands, while the transition toward the center of the sphere that is associated with the MS is related to a shifting toward lower frequency bands (see Figure 1B; Dor-Ziderman et al., 2013; Carhart-Harris et al., 2014; Paoletti et al., 2020), ultimately leading toward the state of OCS (see Figure 2).

We also posit that the electro-topography of the Selves aligns with a hierarchy of meditation practices, from Focused Attention (FA) to Open Monitoring (OM) to Non-Dual (ND) meditation (Laukkonen and Slagter, 2021).^2^ Meditation provides us an excellent tool for investigating the nature of the Self, especially in the rare states of consciousness in which self-processing changes (Laukkonen and Slagter, 2021). Understanding how meditation modulates neural oscillations^3^ may help elucidate the relationship between these brain oscillations and cognitive processes, as well as the different states of Self they may induce. Accordingly, we will show how these three related meditation practices, namely, FA, OM, and ND, follow a similar pattern as the Selves of increasingly slower frequency bands^4^ approaching the center of the SMC sphere.

Thus, we will attempt to show that hierarchical organization based on the level of consciousness of the three types of the Self, which mirrors the hierarchical organization of meditation practice types, can be electrophysiologically measured through frequency bands. This electrophysiological topographic map of the self, reflecting a gradual shift from faster frequency bands, namely, Gamma (30–70 Hz) and Beta (13–30 Hz), to slower ones, namely, Alpha (8–12 Hz), Theta (4–8 Hz), and eventually Delta (1–4 Hz) as we get closer to the center of the Sphere. Thus, we propose three main hypotheses that can be examined through evidence-based electro-topography as specified below:

### Hypothesis 1: The Narrative Self Primarily Involves Gamma and Beta

Empirical grounding for the first hypothesis is based on the finding that Gamma and Beta bands are often correlated with EFs such as focused attention and cognitive effort (Ward, 2003). Since Gamma and Beta are attributed both to FA and working memory, which is essential for the Narrative Self (Laukkonen and Slagter, 2021), we posit that the Narrative Self will be electrophysiologically related to FA meditation.

In fact, Gamma has been consistently reported to increase during engaging self-referential processing and focused attention in different cognitive tasks (Engel et al., 2001; Fell et al., 2003), both of which are related to the Narrative Self (Mantini et al., 2007; Chen et al., 2008), while a general decrease in this band has been reported during more relaxed states of meditation.

Beta is typically associated with sensorimotor processing (Symons et al., 2016). This is supported by studies that have shown a decrease in Beta during OM (Dor-Ziderman et al., 2013; Faber et al., 2015), Transcendental Meditation (Tomljenoviæ et al., 2016), and following an intensive 3-month meditation retreat (Slagter et al., 2007). Decreased Beta activity might be understood as decreased active processing consistent with the traditional understanding that Beta activity is associated with a more active and aroused brain-processing state (Slagter et al., 2009). The findings regarding this frequency band mirror those regarding Gamma frequencies: increasing in relation to the Narrative Self state and decreasing in more relaxed states. Thus, our hypothesis that the Narrative Self is related to FA meditation (Laukkonen and Slagter, 2021), is centered on the notion that both will elicit primarily Gamma and Beta frequencies.

### Hypothesis 2: The Minimal Self Primarily Involves Alpha and Theta

Empirical grounding for the second hypothesis is centered around findings that Alpha and Theta frequencies have been found to increase in more relaxed states, lower arousal, in association with meditation, and with an internally directed focus of attention (Cooper et al., 2003; Takahashi et al., 2005; Lomas et al., 2015; Cona et al., 2020), all of which are related to the Minimal Self (see Section “The Sphere Model of Consciousness, Hierarchy of Selves, and Executive Functions” above). Many studies have found increased Alpha to be associated with meditation, such as Zen and Transcendental Meditation (Kasamatsu and Hirai, 1966; Wallace et al., 1971; Banquet, 1973; Murata et al., 1994, 2004; Yamamoto et al., 2006; see also Baerentsen et al., 2001 for a possible explanation of frontal cortical-subcortical system dominance in Zen meditation initiation).

There are different possible reasons for this relationship between Alpha frequency and meditation. DeLosAngeles et al. (2016) suggested that the widely reported increased Alpha power in meditation could be interpretable as a marker of practice or ease in task performance rather than specific to meditation. Beauregard et al. (2009) have instead suggested that higher frontal and temporal Alpha during meditation is an index of reduced cortical arousal associated with a relaxation response. In addition, as Kerr et al. (2013) beautifully described in their review, with somatic focus, mindfulness’ top-down alpha rhythm modulation “*enhances gain control which, in turn, sensitizes practitioners to better detect and regulate when the mind wanders from its somatic focus*” (pp. 1), a feature fundamentally associated with the Minimal Self. In turn, enhanced regulation of somatic mind-wandering may be an important early stage of mindfulness training that leads to enhanced cognitive regulation and metacognition.

While both OM and FA are related to increased frontal Alpha amplitude and synchrony (Travis, 2001), a recent study reported that OM resulted in an increase in Alpha power, compared to FA, to meditation naïve controls (Himalayan Yoga), and to mind wandering (Braboszcz et al., 2017). There is also evidence that experienced meditators have increased prefrontal and parietal Alpha power during sleep (Dentico et al., 2016; regarding the sleep-wakefulness continuum, see also subsection The Sleep-Wakefulness Continuum below). One reason for this difference between OM and FA is that the top-down regulation of alpha is considered a key mechanism by which advanced OM practitioners learn to disengage attention in order to maintain greater attentional flexibility (Kerr et al., 2013). Alpha is not the only frequency that seems to be particularly related to OM; frontal and parietal Theta coherence have been found to increase in particular during this type of meditation (Cahn et al., 2013).

In addition, both Alpha and Theta are closely related to executive functions such as working memory (Sauseng et al., 2005a). Alpha frequency also plays a critical role in inhibition (Klimesch et al., 2007; Klimesch, 2011) and cognitive flexibility (Fink et al., 2009; Wolff et al., 2017), the other two main components of executive functions.

Theta is also associated with other relevant findings. Notably, Sugimura et al. (2021) reported that participants who tested well in having a strong sense of who they are (i.e., identity synthesis) exhibited increased Theta waves with low noise contamination in their frontal lobe, while those who reported feelings of a temporally changeable and fragmented self (i.e., identity confusion) displayed more Beta waves with high noise interference in the centroparietal lobe. In addition, frontocentral Alpha waves correlated negatively with identity confusion, and frontal Theta waves showed positive relationships with identity synthesis (Sugimura et al., 2021). Additionally, Aftanas and Golocheikine (2001) suggest that Theta is related to thoughtless awareness and bliss.

Taken together, these findings are particularly significant for linking the increase in Alpha and Theta bands to the phenomenology of the Minimal Self. A stronger sense of oneself is, in fact, reported during the Minimal Self state, which is characterized by bodily perception and a more consistent and continuous self-perception compared to the Narrative Self. Moreover, presuming that the Minimal Self is in alignment with OM meditation, both should elicit Alpha and Theta frequencies in particular.

### Hypothesis 3: Overcoming of the Self Primarily Involves Delta Frequency

Empirical grounding for the third hypothesis centers on the finding that Delta is the main frequency associated with non-dual meditative states and higher states of consciousness (Berman and Stevens, 2015). In fact lower frequency bands, namely, Delta (for a recent review, see Wahbeh et al., 2018), are related to meditation’s “core,” and thus are depicted toward the center of the SMC sphere (Paoletti and Ben-Soussan, 2020). This leads us to the third hypothesis that Delta should be primarily associated with Overcoming of the Self (see Section “The Sphere Model of Consciousness, Hierarchy of Selves, and Executive Functions”), and that Non-Dual meditation, which we theorize corresponds with the OCS, should also be particularly associated with Delta.

Delta is reported to increase in deep meditation, especially with higher states of consciousness (Mason et al., 1997; Parker et al., 2013; Berman and Stevens, 2015; Parker, 2017). Unusual Delta activity generated in deep meditation is affiliated with non-conceptual awareness; it may enhance the capacity to suddenly recognize complex, subtle informational patterns that serve to provide novel, relevant solutions to complex problems through insight (Horan, 2009). Consistent with our current hypothesis and supported by previous meditation research Berman and Stevens (2015) found increased Delta, Theta, and Alpha activity during meditation (for a review, see Ivanovski and Malhi, 2007; Wahbeh et al., 2018).

In addition, combining hypothesis 1, 2, and 3 demonstrating a gradual slowing of the frequencies as we approach the center of the SMC sphere, we can also observe a differentiation between general meditation and non-dual states (in which the participant transcends the separation between self and other). The opposite trend was observed for Gamma activity, which was higher during the meditation sessions compared to non-dual states (Berman and Stevens, 2015). Similarly, Berkovich-Ohana et al. (2012), who examined participants who possessed one of three levels of mindfulness expertise versus non-experienced controls, found that mindfulness practitioners generally exhibited reduced resting-state frontal low Gamma power as compared to controls. They also found decreased resting-state Gamma functional connectivity—representing Default Monde Network (DMN) deactivation—among the long-term practitioners, suggesting a trait or long-lasting effect of reduced mind-wandering and self-related processing that is generally associated with the Narrative Self (Berkovich-Ohana et al., 2012, 2013). In addition, creativity, as measured by ideational fluency and flexibility, which was higher among the long-term practitioners than the short-term practitioners and control participants, was negatively correlated with Gamma interhemispheric functional connectivity (Berkovich-Ohana et al., 2017). Thus, one should keep in mind that different meditation techniques can produce different electrophysiological results, depending, among other things, on the extent of experience of the practitioner, and the experimental design (Berman and Stevens, 2015).

In sum, recent electrophysiological studies of meditation consistently demonstrate increased slower frequency bands, and either decreased DMN activation or measures of enhanced sensory, and attentional processing with concomitantly decreased automated reactivity (for a review, see Britton et al., 2014) which could be integrated in the SMC and the hierarchy of Selves. See Table 1 for our mini-review linking different meditation practices and the phenomenological description of the Selves to the electrophysiological findings. The literature review presented in Table 1 was conducted in order to examine our hypothesis that there is a hierarchical order of the electrophysiological correlates that corresponds with the hierarchical order of the three types of the selves and related meditation practices.

**TABLE 1:** Synthesis table summing our mini-review linking different meditation practices and the phenomenological description of the Selves to the electrophysiological findings.

	Meditation	Self	Results			Meditation	Self	Results			Meditation	Self	Results	
									
**Gamma**	OM	**NS**	**+**	Berkovich-Ohana et al., 2012	**Alpha**	Multiple	NS	\	Marzetti et al., 2014	**Theta**	OM/FA*	NS	+	Lutz et al., 2009
	Multiple	**NS**	**+**	Berman and Stevens, 2015							FA	NS	\	Rodriguez-Larios and Alaerts, 2021
	Multiple	**NS**	**+**	Hinterberger et al., 2014		FA	NS	\	Rodriguez-Larios and Alaerts, 2021		FA	**MS**	**+**	Aftanas and Golosheykin, 2005
	Multiple	**NS**	**+**	Lehmann et al., 2001							FA	**MS**	**\**	Baijal and Srinivasan, 2010
	Review	**NS**	**+**	Lomas et al., 2015		FA	**MS**	**+**	Aftanas and Golosheykin, 2005		OM/FA*	**MS**	**+**	Cahn et al., 2010
	Neurofeedback	**NS**	**\**	Van Lutterveld et al., 2017		FA	**MS**	**+**	Banquet, 1973		FA	**MS**	**+**	DeLosAngeles et al., 2016
	OM/FA*	MS	+	Cahn et al., 2010		FA	**MS**	**+**	DeLosAngeles et al., 2016		Multiple	**MS**	**+**	Dunn et al., 1999
	FA	MS	-	DeLosAngeles et al., 2016		Multiple	**MS**	**+**	Dunn et al., 1999		Review	**MS**	**+**	Fell et al., 2010
	LKM	MS	+	Lutz et al., 2004		OM	**MS**	**+**	Faber et al., 2015		OM	**MS**	**+**	Kubota et al., 2001
						Review	**MS**	**+**	Fell et al., 2010		Review	**MS**	**+**	Lomas et al., 2015
	ND	OTS	+	Flor-Henry et al., 2017		OM	**MS**	**+**	Kasamatsu and Hirai, 1966		OM	**MS**	**+**	Murata et al., 1994
						OM	**MS**	**\**	Kerr et al., 2013		OM	**MS**	**\**	Rodriguez-Larios et al., 2020
	ND	OTS	+	Huels et al., 2021		Review	**MS**	**+**	Lomas et al., 2015		OM	**MS**	**+**	Takahashi et al., 2005
						OM	**MS**	**+**	Murata et al., 1994		Multiple	OTS	+	Berman and Stevens, 2015
	FA	OTS	-	Travis et al., 2010		OM	**MS**	**+**	Murata et al., 2004		ND	OTS	+	Flor-Henry et al., 2017
						OM	**MS**	**\**	Rodriguez-Larios et al., 2020		Multiple	OTS	-	Hinterberger et al., 2014
**Beta**	Multiple	**NS/MS**	**+**	Dunn et al., 1999		OM	**MS**	**+**	Takahashi et al., 2005		OM	OTS	+	Winter et al., 2020
	FA	MS	-	DeLosAngeles et al., 2016		Review	**MS**	**+**	Wahbeh et al., 2018	**Delta**	OM/FA*	MS	-	Cahn et al., 2010
	OM	MS	-	Faber et al., 2015		FA	**MS**	**+**	Wallace et al., 1971		Multiple	MS	+	Dunn et al., 1999
	FA	MS	-	Travis et al., 2010		FA	**MS**	**+**	Yamamoto et al., 2006		OM	MS	+	Faber et al., 2008
						Multiple	OTS	+	Berman and Stevens, 2015		Multiple	MS	\	Lehmann et al., 2012
	ND	OTS	+	Flor-Henry et al., 2017		ND	OTS	+	Flor-Henry et al., 2017		FA	MS	\	Tei et al., 2009
						Multiple	OTS	-	Hinterberger et al., 2014		Multiple	**OTS**	**+**	Berman and Stevens, 2015
	Multiple	OTS	-	Hinterberger et al., 2014		OM	OTS	-	Lo et al., 2003		ND	**OTS**	**+**	Flor-Henry et al., 2017
						FA	OTS	+	Travis et al., 2010		Multiple	**OTS**	**-**	Hinterberger et al., 2014
	Multiple	OTS	\	Lehmann et al., 2012							ND	**OTS**	**+**	Parker, 2017
						OM	OTS	-	Winter et al., 2020		ND	**OTS**	**+**	Parker et al., 2013

Attribution of self has been done accordingly to the phenomenological description of results done by authors of cited papers. Main hypotheses are colored in blue scale (with increasingly dark blue color as the findings relate toward the center of the sphere). Highlighted cells are findings related to the main hypotheses of the proposed model. Increase: +; Decrease: -; Changes in connectivity or Neurofeedback: *˙Vipassana meditation (VM) can be understood as a combination of FA meditation and OM meditation (Lutz et al., 2008).

## Discussion on the Model and Current Hypothesis

Of the articles reviewed, 83% support our model of the electro-topography of the Selves with increasingly slower frequency bands as one approaches the center of the SMC sphere (Table 2).

**TABLE 2:** Numerical summary of literature mini-review findings.

Agreement with hypotheses		
**Papers**	***N***	**% of agreement with main hypotheses**

Papers regarding main hypotheses	24	83%
Papers in agreement with main hypotheses	20	

*Number and percentage of studies in agreement with our hypotheses. Reviews were not counted, and papers in agreement with hypotheses for more than one frequency were only counted once. Papers were considered “relevant to main hypotheses” if they examined the hypothesized frequency in association with a phenomenological description fitting the hypothesized-as-correlated Self state. The direction of the findings was then evaluated to see if it agreed with the hypotheses.*

To further support our hypothesis, we will now also present a number of our previous study results regarding the relationship between frequency bands and self-awareness through: (1) Mindfulness meditation, (2) Quadrato Motor Training (QMT), and (3) The Whole-Body Perceptual Deprivation chamber (OVO-WBPD) (Table 3). The last two are techniques that were created from the SMC itself. The results support the relationship between electrophysiological frequency bands and the SMC. More specifically:

1. Mindfulness meditation. We previously reported higher fluency and flexibility, which are measures of creativity, among two long-term mindfulness practitioners as compared to short-term mindfulness practitioners and control participants, which were negatively correlated with trait default-mode gamma inter-hemispheric functional connectivity (Berkovich-Ohana et al., 2017). This study indicates that a form of mindfulness meditation that shares similarities with QMT can reduce the role of the DMN and the involvement of gamma frequency band. In terms of the model, mindfulness meditation can promote distancing from Narrative Self and from fast frequency bands like gamma. This is in line with the proposed electro-topography, since processes and networks (DMN) commonly related to the Narrative Self have also been found associated with gamma frequency outside the context of the SMC and the scope of the present paper (e.g., Engel et al., 2001; Laufs et al., 2003a,b; Chen et al., 2008; Berkovich-Ohana et al., 2012).
2. The QMT is a specifically structured movement meditation in which the participants make a step into one of 12 possible directions according to verbal instruction (Paoletti and Salvagio, 2011; Dotan Ben-Soussan et al., 2013). QMT relates mainly to processes associated with the Minimal Self as it requires ongoing second-by-second mindful awareness to the body and to the upcoming command (Ben-Soussan et al., 2019b; Leshem et al., 2020).
3. The OVO-WBPD chamber is an altered sensory environment in the form of a human-sized egg (uovo in Italian literally means “egg”). Based on the SMC, the OVO-WBPD was specifically built with the aim of facilitating an immersive experience and an increased state of presence (Paoletti, 2002a). While the QMT is related more to the Minimal Self, the experience in the OVO-WBPD is more related to the Overcoming of the Self, since it induces a state of absorption and dissolves spatial boundaries (Ben-Soussan et al., 2019a).

**TABLE 3:** Summary of studies relating Quadrato Motor Training (QMT), OVO Whole Body Perceptual Deprivation chamber (OVO-WBPD), and mindfulness meditation to different states of Self and their electrophysiological correlates.

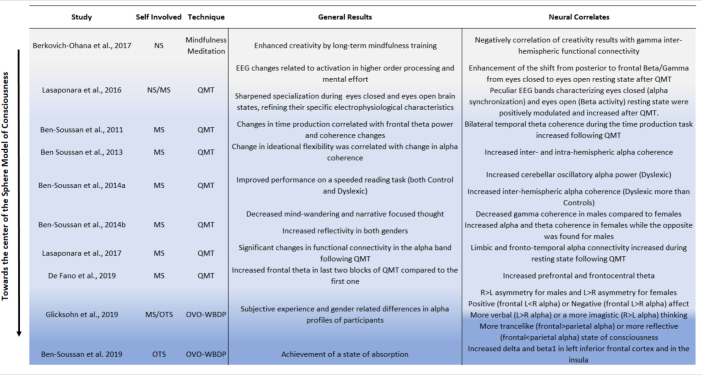

*Main hypotheses are colored in blue scale (with increasingly dark blue color as the findings relate toward the center of the sphere).*

Taken together, these techniques and their electrophysiological correlates (shown in Table 3) suggest a gradual shift from the Narrative Self and higher frequency bands toward the Minimal Self and then toward Overcoming of the Self and lower frequency bands that we are proposing with the present mini-review.

In Table 4, we also highlight the spatial localization of the EEG results reported in this mini-review. It is possible to see that Alpha and Theta frequency bands display the most consistent results with predominant frontal activity, while Gamma and Delta frequency bands are less spatially localized. One possible explanation for these results could be the scale-dependent mechanism highlighted by Von Stein and Sarnthein (2000) for visual processing. The authors proposed that the more local the synchronization, the higher the frequency involved. In particular, they showed that “local interactions during visual processing involve gamma frequency dynamics, semantical interactions between temporal and parietal cortex involve beta frequency dynamics, and very long-range interactions […] a low theta or alpha frequency range.”

**TABLE 4:** Summary of main studies and their localization.

	Meditation	Self	Results		Area localization
	
**Gamma**	OM	**NS**	**+**	Berkovich-Ohana et al., 2012	Parieto-Occipital
	Multiple	**NS**	**+**	Berman and Stevens, 2015	Frontal, Central, Parietal, Temporal, and Occipital
	Multiple	**NS**	**+**	Hinterberger et al., 2014	Central and Parietal
	Multiple	**NS**	**+**	Lehmann et al., 2001	Frontal and Temporo-Parietal
	Neurofeedback	**NS**	**\**	Van Lutterveld et al., 2017	PCC
	OM/FA	**MS**	**+**	Cahn et al., 2010	Parieto-Occipital
	FA	**MS**	**-**	DeLosAngeles et al., 2016	Central
	LKM	**MS**	**+**	Lutz et al., 2004	Frontal and Temporo-Parietal
	ND	**OTS**	** +**	Flor-Henry et al., 2017	Parietal
	ND	**OTS**	**+**	Huels et al., 2021	N/A
	FA	**OTS**	**-**	Travis et al., 2010	Frontal and Temporal
**Beta**	Multiple	**NS/MS**	**+**	Dunn et al., 1999	Central and Parietal
	FA	**MS**	**-**	DeLosAngeles et al., 2016	Central
	OM	**MS**	**-**	Faber et al., 2015	Central and Parietal
	FA	**MS**	**-**	Travis et al., 2010	Frontal and Parietal
	ND	**OTS**	**+**	Flor-Henry et al., 2017	Frontal
	Multiple	**OTS**	**-**	Hinterberger et al., 2014	Parietal
	Multiple	**OTS**	**\**	Lehmann et al., 2012	Central
**Alpha**	Multiple	**NS**	**\**	Marzetti et al., 2014	PCC
	FA	**NS**	**\**	Rodriguez-Larios and Alaerts, 2021	N/A
	FA	**MS**	**+**	Aftanas and Golosheykin, 2005	Frontal
	FA	**MS**	**+**	Banquet, 1973	Frontal
	FA	**MS**	**+**	DeLosAngeles et al., 2016	Frontal and Temporo-Parietal
	Multiple	**MS**	**+**	Dunn et al., 1999	Central and Parietal
	OM	**MS**	**+**	Faber et al., 2015	Frontal and Temporal
	OM	**MS**	**+**	Kasamatsu and Hirai, 1966	Frontal
	OM	**MS**	**\**	Kerr et al., 2013	Central
	OM	**MS**	**+**	Murata et al., 1994	Frontal
	OM	**MS**	**+**	Murata et al., 2004	Frontal
	OM	**MS**	**\**	Rodriguez-Larios et al., 2020	N/A
	OM	**MS**	**+**	Takahashi et al., 2005	Frontal
	FA	**MS**	**+**	Wallace et al., 1971	Frontal
	FA	**MS**	**+**	Yamamoto et al., 2006	Frontal
	Multiple	**OTS**	**+**	Berman and Stevens, 2015	Frontal, Central, Parietal, Temporal, and Occipital
	ND	**OTS**	**+**	Flor-Henry et al., 2017	Frontal, Temporal, Parietal and Occipital
	Multiple	**OTS**	**+**	Hinterberger et al., 2014	Parietal
	OM	**OTS**	**-**	Lo et al., 2003	N/A
	FA	**OTS**	**+**	Travis et al., 2010	Frontal and Central
	OM	**OTS**	**-**	Winter et al., 2020	Parietal
**Theta**	OM/FA	**NS**	**+**	Lutz et al., 2009	Frontal
	FA	**NS**	**\**	Rodriguez-Larios and Alaerts, 2021	N/A
	FA	**MS**	**+**	Aftanas and Golosheykin, 2005	Frontal
	FA	**MS**	**\**	Baijal and Srinivasan, 2010	Frontal Increase, Parietal Decrease
	OM/FA	**MS**	**+**	Cahn et al., 2010	Frontal
	FA	**MS**	**+**	DeLosAngeles et al., 2016	Frontal and Temporo-Parietal
	Multiple	**MS**	**+**	Dunn et al., 1999	Frontal, Central, Parietal, Temporal, and Occipital
	OM	**MS**	**+**	Kubota et al., 2001	Frontal
	OM	**MS**	**+**	Murata et al., 1994	Frontal
	OM	**MS**	**\**	Rodriguez-Larios et al., 2020	N/A
	OM	**MS**	**+**	Takahashi et al., 2005	Frontal
	Multiple	**OTS**	**+**	Berman and Stevens, 2015	Frontal, Central, Parietal, Temporal, and Occipital
	ND	**OTS**	**+**	Flor-Henry et al., 2017	Frontal and Temporo-Parietal
	Multiple	**OTS**	**-**	Hinterberger et al., 2014	Frontal, Central, Parietal, Temporal, and Occipital
	OM	**OTS**	**+**	Winter et al., 2020	Frontal, Central, Parietal, and Occipital
**Delta**	OM/FA	**MS**	**-**	Cahn et al., 2010	Frontal
	Multiple	**MS**	**+**	Dunn et al., 1999	Frontal and Parietal
	OM	**MS**	**+**	Faber et al., 2008	PFC
	Multiple	**MS**	**\**	Lehmann et al., 2012	Frontal and Parietal
	FA	**MS**	**\**	Tei et al., 2009	Frontal Increase; Central, Parietal, and Temporal Decrease
	Multiple	**OTS**	**+**	Berman and Stevens, 2015	Frontal, Central, Parietal, Temporal, and Occipital
	ND	**OTS**	**+**	Flor-Henry et al., 2017	Temporal
	Multiple	**OTS**	**-**	Hinterberger et al., 2014	Frontal, Central, Parietal, Temporal, and Occipital
	ND	**OTS**	**+**	Parker, 2017	N/A
	ND	**OTS**	**+**	Parker et al., 2013	N/A

*Reviews were excluded from this table. Not applicable (N/A) are studies without a clear or not reported localization of results. Main hypotheses are colored in blue scale (with increasingly dark blue color as the findings relate toward the center of the sphere).*

Although these conclusion concern visual processing, they appear highly relevant because they are consistent with the findings reported in Table 4 and they offer a cogent explanation as to why the Delta frequency band is more globally distributed than other “faster” frequencies. In addition, Von Stein and Sarnthein (2000) stated that Alpha and Theta frequencies seem to be “specifically involved in processing of internal mental context” which is in line with the role that these frequency bands have in the proposed electro-topography model and in the previous literature (Takahashi et al., 2005; Jensen and Mazaheri, 2010; Klimesch, 2012; Benedek, 2018; Cona et al., 2020), further supporting our hypothesis.

### Suggested Hypothesis Testing and Application

#### First-Person Phenomenology and Self-Awareness

Probably the biggest challenge in the study of consciousness is how to deal with the first-person subjective experience within an objective measurable framework. According to the SMC, all phenomenal characteristics of experience can be placed along three axes: Time, Emotion, and Self-Determination (see Figure 1; Paoletti, 2002a; Paoletti and Ben-Soussan, 2019, 2020, 2021). The highest degree of self-awareness can be represented as an equal relationship between the periphery of the sphere, or the extremes of the axes on which the features of first-person experience are placed, and the center, where we place consciousness-as-such/non-dual consciousness. While the spherical matrix of the model provides a diagram for representing first-person experience, our hypotheses on the electro-topography of the Selves might enable us in future studies to find neurophysiological correlates for first-person experience. For instance, recalling Raffone et al. (2019) argument about the Beta frequency (the possibility of different levels or grades of self-awareness within each dimension of Self), we might observe this frequency in a state of self-projection as well in FA meditation with good self-awareness.

Our model may also serve as a guideline to refine the research on correlates to self-awareness phenomenology: when Beta is present, for example, we hypothesize a connection with the Narrative Self, but can we also detect differences in its coherence, amplitude, and localization in specific brain areas? What would that imply? For example, can we detect electrophysiological brain correlates in areas suggested in different models as the main areas for consciousness correlation–like Broadman Area 10 (Raffone and Srinivasan, 2009) or the precuneus (Josipovic, 2019)–according to the Self we are observing?

Since several works have already been published on the topic, we will only focus briefly on the problem of ineffability (see Section “The Problem of Ineffability”), and then present several difficulties that have not (with few exceptions) been sufficiently addressed yet in the research literature, such as the sleep-wakefulness continuum (see also Section “The Sleep-Wakefulness Continuum”) and the distinction between higher and altered states of consciousness (see Section “The Importance of Differentiating Between Altered and Higher States of Consciousness”). As detailed above (Section “The Sphere Model of Consciousness, Hierarchy of Selves, and Executive Functions”), the Minimal Self and the Narrative Self appear to be more directly related to executive functions (EFs), while the Overcoming of the Self presents us with the problem of ineffability, which will now be shortly addressed.

#### The Problem of Ineffability

In order to electrophysiologically and behaviorally map the different Selves, we need to distinguish between consciousness and its contents and, consequently, deal with the challenge of ineffability. One of the main obstacles in studying and quantifying higher states of consciousness is the ineffability of subjective experiences. When one’s sense of self “disappears,” as is reported in first-person accounts of the Overcoming of the Self, we encounter something hardly measurable by the same behavioral and cognitive parameters used to assess other states of the Self which are mostly identified with/described by the processes involved (e.g., Narrative Self is associated with autobiographical processes and conceptual contents; Gallagher, 2000; Oatley, 2007). Yet, taking neurophenomenological studies for reference, we can electrophysiologically measure whether consciousness-without-content or non-dual awareness is in line with some of the correlates for that state, as suggested by the most recent models of consciousness proposed by Raffone and Srinivasan (2009); Metzinger (2018), and Josipovic (2019). These models conceptualize consciousness-without-content, non-dual awareness, and the Overcoming of the Self (in the case of the SMC) which can be easily measured using EEG. Indeed, various attempts are being made to do so (Berkovich-Ohana et al., 2017). For this reason, the hypothesized electrophysiological model could provide an interesting index (i.e., increase in Delta frequency band) to identify the state in which any sense of self disappears and reportable cognitive processes and behavioral measures become less informative.

#### The Sleep-Wakefulness Continuum

Another possible application for the proposed electro-topographic model is in the examination and differentiation of various states of wakefulness. More specifically, the sleep-wakefulness continuum is often treated as dichotomous, but various cultures have described different states of awareness outside this dichotomy (Walsh and Vaughan, 1993). One of these states is the outcome of transcendent meditation: a deeply restful state but with fully alert inner wakefulness (Alexander and Sands, 1993). Utilizing this state of consciousness as an example of an “in-between state” along the sleep-wakefulness continuum, we will apply our proposed model to explore if the proposed electro-topography matches the electroencephalography findings in the literature.

Further, considering the phenomenological description of transcendental consciousness as a profound state of relaxation but with preserved awareness, and following the SMC-based hypotheses presented in this paper, we postulate that this state of consciousness will be found associated with increased activity in Alpha and Theta bands (Wallace et al., 1971; Banquet, 1973; Mason et al., 1997; Yamamoto et al., 2006).

For example, Theta plays important role in memory encoding and consolidation during sleep (for a review, see Rasch and Born, 2007). Nonetheless, as Kirov et al. (2009) demonstrated, application of transcranial slow oscillation stimulation (tSOS) can enhance the process of memory consolidation not only during sleep (Marshall et al., 2006), but also in wakeful states. This enhancement was accompanied by a widespread increase of Theta activity (Kirov et al., 2009). Thus, the hypothesized correlation between the Theta frequency and the Minimal Self could help in understanding differences in states of awareness along the sleep-wakefulness continuum, and to further expanding its currently dichotomous definition into more subtle distinctions between different wakefulness states.

#### The Importance of Differentiating Between Altered and Higher States of Consciousness

So far, we have demonstrated how the hypothesized model is in alignment with different meditation traditions. In addition, we further suggested that the current hypothesized electro-topography could be useful in guiding new contemplative neuroscience investigations into differentiating Altered States (AS) from Higher States (HS) of consciousness. Altered States (AS) are traditionally defined as “*a qualitative alteration in the overall pattern of mental functioning, such that the experiencer feels consciousness radically differently from the ‘normal’ way it functions. It should be noted that an AS is not defined by a particular content of consciousness, behavior, or physiologic change, but in terms of overall patterning.*” (Tart, 1972; for a review on neurobiological basis of AS, see also Vaitl et al., 2005).

However, not all AS are alike. While higher states of consciousness are accompanied by improved executive functionality, the opposite is true for many cases of drug-induced AS. Moreover, electrophysiologically, HS are accompanied by slower frequency bands and are considered more integrated states (Mason et al., 1997), while EEG and MRI studies of drug-induced AS report an opposite trend that could be regarded as fragmentation and consequent deregulation.

Moreover, an important distinction must be made between general AS and HS. According to Tart (1972), higher states, in particular, can be associated with superior cognitive functioning or can be more profound than other states; these states can include insights into oneself, insights into others, intuitive understanding of the nature of the universe, or comprehension of an individual’s place in the overall scheme of things (in relation to insight, see also Section The Sphere Model of Consciousness and Self Are Dynamic below). Although this description is phenomenologically exhaustive, it is not sufficient (as acknowledged by the author) to distinguish “*any unequivocally higher state*” Electrophysiology, therefore, could be a reliable method to reach a more precise discrimination between HS and AS.

Electrophysiological studies and the proposed model can, indeed, help in differentiating between AS and HS of consciousness. Although this is not the aim of the paper, we will focus briefly on the differentiation between contemplative-induced HS of consciousness versus drug-induced AS, in order to provide a preliminary framework. To examine our hypothesis, we reviewed the literature from the perspective of its applicability to SMC electro-topography. We found that the main difference between AS and HS is primarily in Alpha and higher frequencies: HS are consistently related to increased Alpha activity while AS displays reduced Alpha activity in this EEG band (see Table 5). Taken together, in HS, deep states of meditation and attention are achieved through discipline and focus which largely involve EF training. While achievement of an AS is easier, often being driven by consumed substances, there is a lesser degree of control over the whole experience. This lesser degree of intentional and skillful navigation of the experiences could undermine the replicability and increase possible dangers related to drug-induced AS compared to the safety and replicability of HS. A good example for this, would be a professional athlete or dancer that can intentionally and reliably perform difficult and potentially dangerous movements compared to a novice that perform the same actions by chance, risking injuries and without being able to replicate or even know how that happened. This example is also quite fitting to the findings of Fink et al. (2009), who found increased Alpha activity in professional dancers compared to novices during mental creation of dances.

**TABLE 5:** Summary of examined studies regarding Alpha Frequency in Altered States (AS) and Higher States (HS) differentiation.

	Study	State achievement	Alpha
**Altered States of Consciousness**	Fink, 1969	LSD Intake	**+**
	Sannita et al., 1987	Scopolamine Intake	**−**
	Sloan et al., 1992	Scopolamine Intake	**−**
	Neufeld et al., 1994	Scopolamine Intake	**−**
	Ebert et al., 2001	Scopolamine Intake	**−**
	Riba et al., 2002	Ayahuasca Intake	**−**
	Osipova et al., 2003	Scopolamine Intake	**−**
	Riba et al., 2004	Ayahuasca Intake	**−**
	Muthukumaraswamy et al., 2013	Psilocybin Intake	**−**
	Kometer et al., 2015	Psilocybin Intake	**−**
	Carhart-Harris et al., 2016	LSD Intake	**−**
**Higher States of Consciousness**	Travis et al., 2002	Trascendent Meditation	**+**
	Abdullah and Omar, 2011	Religious Contemplation	**+**
	Doufesh et al., 2012	Religious Contemplation	**+**
	Doufesh et al., 2014	Religious Contemplation	**+**
	Vaghefi et al., 2015	Religious Contemplation	**+**
	Berman and Stevens, 2015	Multiple types of Meditation	**+**
	DeLosAngeles et al., 2016	Concentrative Meditation	**+**
	Al-Galal and Alshaikhli, 2017	Religious Contemplation	**+**
	Wahbeh et al., 2018	Trascendent Meditation	**+**
	Barcelona et al., 2020	Religious Contemplation	**+**

To sum up, HS could be linked to increased Alpha activity because these states entail effort and inhibition in controlling the process, while AS could be related to disinhibition and loss of control as reflected by overall decreased Alpha activity. This could be explained by the proposed electro-topography model as the result of increased internally directed attention and embodiment (Minimal Self) during the process of achieving HS, which is lacking during a drug-induced AS.

## Limitations and Considerations in the Interpretation of the Model

Despite the supporting evidence, there are limitations to the proposed model that should be acknowledged. First, the electrotopographic model may seem oversimplified because, as depicted, the examined frequency bands often underlie a wide range of different cognitive functions. However, we are not suggesting that a given meditation type, for instance, is exclusively associated with one frequency band’s activity, but rather we hypothesize that a specific meditation type relies more on that frequency than others. Of necessity, the graphic depiction of this requires some simplification.

In addition, there are two patterns of results that apparently contradict to our hypothesis. First, regarding our hypothesis that the Minimal Self primarily involves Alpha and Theta frequencies, paradoxically, some findings in the literature have reported increased Alpha and Theta activity during mind-wandering (Rodriguez-Larios et al., 2020; Rodriguez-Larios and Alaerts, 2021), which is related to the Narrative Self. However, this could also be attributed to retrieval and manipulation of memory information (Bastiaansen et al., 2002; Jensen et al., 2002; Sauseng et al., 2002, 2004, 2005a,2005b; Moran et al., 2010; see also Staresina and Wimber, 2019 for a review). Second, increased Gamma/decreased Delta during deep meditation could be related to the central role that Gamma plays in brain mechanisms underlying information processing (Lehmann et al., 2001). Increased Gamma activity could also be related to the spiritual/religious/mystic aspects of the meditative state (Beauregard et al., 2009) and therefore to higher states of consciousness with fullness of content, while lower frequency bands (Delta, Theta, and Alpha) could be related to states of thoughtless emptiness (meaning higher yet content-less states). Perhaps further discrimination between “deeper” states, taking into consideration not only the level of consciousness involved but also the content of those states, would be worthwhile to explore in future research.

A further aspect to consider in future research is the strong relationship among phenomenological experiences, EFs, and electrophysiology that have been reported across many studies exploring the concept of Self. Future studies should explore these relationships in a systematic way, taking into consideration different styles of meditation (see Gallant, 2016 for a review examining EFs with regard to mindfulness meditation).

What is more, as mentioned in the Introduction, the subjective dimension of self-awareness remains inescapable. For instance, based on Raffone et al. (2019) review, we can argument about the possibility of different levels or grades of self-awareness within each dimension of Self, namely, we might observe Beta frequency in a state of self-projection as well in FA meditation with good self-awareness.

### The Sphere Model of Consciousness and Self Are Dynamic

The proposed model is dynamic in several noteworthy ways. As the Self is dynamic, so might one oscillate between meditative states during meditation practice. For example, movement between FA and OM often occurs (Laukkonen and Slagter, 2021). In the same way, mechanisms and functions underlying the Self could also be dynamic: an active and intentional effort could dynamically restructure self-awareness, consciousness, and EFs in different ways. Indeed, two of the most studied phenomena that share some form of self-awareness and restructuring with meditation are cognitive reappraisal^5^ and insight.^6^

Many studies identify an association between Gamma and the process of achieving deeper states of meditation, as well as with the degree of visual imagery involved in these states. In particular, the Gamma band could be associated with the hyper-focused attention required to *achieve* non-dual states, but not with the non-dual state itself (Berman and Stevens, 2015). This could be due to the relationship between Gamma, and meditation, cognitive reappraisal, and insight^7^ (Jung-Beeman et al., 2004): These processes rely on a restructuring of awareness producing similar effects (e.g., sudden and clear solutions; new perception of emotional stimuli). However, these could also be reached through different mechanisms: active and intentional efforts to solve a problem rely, at least partly, on executive functions (Sandkühler and Bhattacharya, 2008; Schmeichel et al., 2008). Meditation, in contrast, may rely on deeper and more self-less states that involve less conceptual processing.

From an electrophysiological point of view, as discussed earlier, different investigations have found decreased prefrontal Alpha activity during reappraisal (Parvaz et al., 2012; Choi et al., 2016; Li et al., 2021) in association with increased prefrontal Theta activity (Ertl et al., 2013). Possible generators of frontal Theta oscillations have been suggested to reside in the medial prefrontal cortex (PFC)/anterior cingulate complex (ACC) (Pizzagalli et al., 2002; Mulert et al., 2007a,b). However, although the ACC is notably one of the areas most associated with insight (Dietrich and Kanso, 2010), it is thought to prompt weak, subconscious solutions (Kounios and Beeman, 2009).

What could be the reason for these similarities in brain activity? One reason for shared areas and electrophysiological correlates is that both cognitive reappraisal and insight have been associated with core EFs, such as working memory processes (Schmeichel et al., 2008). For example, frontal Theta activity in the ACC has been found to increase with task difficulty and memory demands (Gevins et al., 1997; Lazarev, 1998; Kahana et al., 1999; Wilson et al., 1999; Krause et al., 2000; but see Bastiaansen et al., 2002). Successful retrieval is also associated with activity in the Theta band (Weiss and Rappelsberger, 2000; Klimesch et al., 2001; Sederberg et al., 2003). Thus, such similarities could result from the involvement of wider, more global functions that underlie all the Self states.

## Conclusion

In conclusion, to our knowledge, this is the first attempt to systematically integrate electrophysiological accounts with different Selves and meditation practices. Our core proposal is that there is an electro-topographic hierarchy of the Selves that mirrors the hierarchy of meditation types. We have shown a parallel between FA/OM/ND and NS/MS/OCS in which Gamma and Beta = the Narrative Self and Focused Attention meditation; Alpha and Theta = the Minimal Self and Open Monitoring meditation; and Delta = Overcoming of the Self and Non-Dual meditation. Our evidence should be viewed as preliminary since these are complex processes that involve many different areas, networks, and frequency bands all at once. Future studies should examine the parallel between the electrophysiological change and the neurophenomenological shift between Selves, employing different training paradigms that utilize whole brain analysis of different frequency bands.

Moreover, we also highlighted how the frequency bands involved in both hierarchies are associated with EFs. Future behavioral and electrophysiological studies should aim to assess how EFs in general, and their components in specific, could play a critical role in structuring the Self and in the switching between different states (for a very recent review on mapping EFs and their components using electrical subcortical stimulation, see Landers et al., 2021).

Additionally, given the aforementioned role of neuronal noise in “identity confusion” (Sugimura et al., 2021) and the lack of it in feelings of identity consistency and integration, future studies should also explore possible parallels between phenomenological and electrophysiological silence, which is a very common feature in meditative practices (for empirical and theoretical contributions to neural, psychological, and contemplative correlates of silence, see Ben-Soussan et al., 2021).

Although a comprehensive electrophysiological model encompassing all frequency bands and their dynamics—including detailed temporal and spatial localization evidence—is still far from being achieved, there are relevant findings in recent literature that offer potential direction for future studies. For example, a recent work of Rodriguez-Larios et al. (2020) examined the Alpha-Theta cross-frequency coupling and considered these two frequency bands and their dynamics during meditation. This is particularly interesting because authors not only examined Alpha and Theta bands from the point of view of a linear increased/decreased activity, but also how reciprocal changes in these frequencies’ activity (especially their ratio) could facilitate meditation training. Such dynamics were also explored in the field of neurofeedback, in which the importance of the Alpha/Theta ratio in relation to creativity and artistic performance was emphasized (Gruzelier, 2009; Gruzelier et al., 2014a,b). These findings may offer directions for expanding and refining our proposed electro-topography. Moreover, these directions should orient and guide the field of consciousness studies and contemplative neuroscience, which should systematically start reporting all the different frequency bands and to integrate evidence concerning electroencephalographic dynamics into the various models that currently exist.

In summary, based on the presented evidence, we suggest that the Narrative Self may be related to reduced self-awareness in the “here and now” compared to the Minimal Self that is more mindful. Further, shifting between the Narrative Self to the Minimal Self correlates with an increase in self-awareness which, in turn, correlates with slower frequency bands, namely, Alpha and Theta oscillation (Paoletti and Ben-Soussan, 2020). The third state of Overcoming of the Self correlates with increasingly slower frequency bands, namely, Delta. Similarly, different kinds of meditations align with the same electrophysiological hierarchy. However, this does not necessarily imply that one meditation is better than another in enhancing self-awareness, only that there is a gradual shift in self-awareness’ phenomenology (Laukkonen and Slagter, 2021).

Our proposed model may aid in resolving some inconsistencies between different frequency bands and locations pointed to in previous studies as the main correlates of consciousness and meditative practices. According to our proposal, these different frequency bands and locations may result from the different attentional states and related Selves that were examined. The model can be tested and applied in different contexts such as in the examination of advanced meditative states, higher states versus altered states, and levels of wakefulness. Accordingly, this new electro-topographic framework of the Self and meditation may more easily facilitate greater understanding of the connections between them, with implications for research in wakefulness states, altered states of consciousness, and executive functions.

## Author Contributions

PP was the creator of the Sphere Model of Consciousness and contributed parts related to the neuro-psychological applications of his model. TB-S mostly contributed to the electro-topographic map of the self. RL mostly contributed parts related to the executive functions. MP conducted the mini-review. All authors contributed to the article hypothesis, writing, and approved the submitted version.

## Conflict of Interest

The authors declare that the research was conducted in the absence of any commercial or financial relationships that could be construed as a potential conflict of interest.

## Publisher’s Note

All claims expressed in this article are solely those of the authors and do not necessarily represent those of their affiliated organizations, or those of the publisher, the editors and the reviewers. Any product that may be evaluated in this article, or claim that may be made by its manufacturer, is not guaranteed or endorsed by the publisher.

## Footnotes

1 In relation to this, in certain meditation practices, attention must eventually be released in favor of a form of bare or non-preferential attention, while in non-dual meditation practices, attention is released altogether (Dunne, 2011) as it claims to employ reflexive awareness that permits non-dual witnessing of the subject-object dichotomy and results in a pure (non-dual) awareness. In fact, according to some traditions, our inability to ordinarily detect non-dual awareness “is due to an obscuration of this reflexive property by mistaken cognitions arising from substrate consciousness. Although NDA [non-dual awareness] is experienced in meditation as a vivid presence of empty awareness that knows Itself directly without mediation by conceptual thought, substrate consciousness is experienced as a pleasantly restful absorbed state, akin to deep sleep yet not entirely unconscious” (Josipovic, 2014, pp. 2). In addition, the voluntary “letting go” in “states of flow,” a transitory state between the MN and OCS, is related to altered states of consciousness (ASC). This deliberate reduction in the use of executive processes can also occur in the experience of flow (Csikszentmihalyi, 2000). Flow may result from a voluntary act occurring in both meditation and sports, a tuning in that involves “letting go” of goal (Jackson and Csikszentmihalyi, 1999; Lutz, 2009; Brown and Leary, 2016; Paoletti et al., 2020). It is important to note that alpha activity has been found to be related to the experience of flow (Katahira et al., 2018) and considering the involvement of alpha frequency both in flow and as main difference between Altered States and Higher States, this voluntary “letting go” could be critical in discriminating between these states of consciousness.

2 Another reason for the similarities between electrophysiological correlates of the electro-topography of the Selves and meditation types could be related to the different executive functions required for each of these states, which may be related to different levels of self-awareness, as we have seen in Section “The Sphere Model of Consciousness, Hierarchy of Selves, and Executive Functions.”

3 In particular, neural oscillations can be evaluated in a local region or between various regions of the brain. The strength of a particular oscillatory frequency in a particular region can be analyzed using a power analysis. Coherence, on the other hand, is the degree of coupling of a particular frequency between two different brain regions and can be used as an indicator of functional connectivity. EEG and MEG can both be used to investigate power and coherence within a particular frequency band.

4 FA meditation requires voluntary focusing attention on a chosen object, OM meditation involves non-reactive monitoring of the content of experience from moment to moment (Lutz et al., 2009), and ND meditation aims for awareness that is unchanging regardless of what happens in experience (Josipovic, 2010; Dunne, 2011; Metzinger, 2020). ND awareness is characterized by its reflexive property—it knows itself to be conscious without relying on conceptual cognition (Josipovic, 2014; Laukkonen and Slagter, 2021). These meditation types also reflect different neural activity. FA, such as in the case of Tibetan Buddhist, Buddhist, and Chinese practices (Travis et al., 2010), has been mostly associated with gamma and beta activity (Hinterberger et al., 2014), OM with theta activity (Lee et al., 2018) and ND with delta activity (Knyazev, 2012; Berman and Stevens, 2015).

5 Reappraisal refers to the act of changing the way one thinks about emotional events (for a review, see John and Gross, 2004). In particular, cognitive reappraisal strategies have been investigated mainly in relation to aversive stimuli (Ochsner et al., 2004; Banks et al., 2007; Kanske et al., 2011; Schulze et al., 2011). This cognitive strategy for emotional regulation has been found to be strongly related to contemplative practices. Garland et al. (2009, 2010, 2011) and Garland and Howard (2013) theorized that mindfulness training can facilitate cognitive reappraisal. Moreover, Hanley et al. (2014) demonstrated that a substantial proportion of practitioners who use a wide range of contemplative practices reportedly engaged in mindful reappraisal techniques as a means of coping with serious stressors or the hassles of everyday life.

6 Insight is a sudden comprehension, the famous “*Eureka!*” that can result in a new interpretation, a reorganization or restructuring of the elements of a situation and can point to the solution to a problem (Sternberg and Davidson, 1995). This definition seems to be very similar to the following description of a sudden transformation occurring during meditation: “*A common experience for long term meditators is that the ‘new’ knowledge being encoded is, paradoxically, the experience of a thought-free state transforming all phenomena into novelty. Phenomena become new because “there is no succession of perception of this knowledge; it takes in all things simultaneously, at a glance*” (Nikhilananda, 1956, pp. 203; from Horan, 2009).

7 This could also explain the difference in Gamma between experienced and novice meditators (i.e., less Gamma = less effort among experienced meditators in achieving a non-dual state). For example, long-term Vipassana meditation contributes to increased occipital Gamma power and is related to long-term meditational expertise and enhanced sensory awareness (Cahn et al., 2010).
